# Path Integral Methods for Stochastic Differential Equations

**DOI:** 10.1186/s13408-015-0018-5

**Published:** 2015-03-24

**Authors:** Carson C. Chow, Michael A. Buice

**Affiliations:** Mathematical Biology Section, Laboratory of Biological Modeling, NIDDK, NIH, Bethesda, MD 20892 USA

## Abstract

Stochastic differential equations (SDEs) have multiple applications in mathematical neuroscience and are notoriously difficult. Here, we give a self-contained pedagogical review of perturbative field theoretic and path integral methods to calculate moments of the probability density function of SDEs. The methods can be extended to high dimensional systems such as networks of coupled neurons and even deterministic systems with quenched disorder.

## Introduction

In mathematical neuroscience, stochastic differential equations (SDE) have been utilized to model stochastic phenomena that range in scale from molecular transport in neurons, to neuronal firing, to networks of coupled neurons, to cognitive phenomena such as decision making [1]. Generally these SDEs are impossible to solve in closed form and must be tackled approximately using methods that include eigenfunction decompositions, WKB expansions, and variational methods in the Langevin or Fokker–Planck formalisms [2–4]. Often knowing what method to use is not obvious and their application can be unwieldy, especially in higher dimensions. Here we demonstrate how methods adapted from statistical field theory can provide a unifying framework to produce perturbative approximations to the moments of SDEs [5–13]. Any stochastic and even deterministic system can be expressed in terms of a path integral for which asymptotic methods can be systematically applied. Often of interest are the moments of $x(t)$ or the probability density function $p(x,t)$. Path integral methods provide a convenient tool to compute quantities such as moments and transition probabilities perturbatively. They also make renormalization group methods available when perturbation theory breaks down. These methods have been recently applied at the level of networks and to more general stochastic processes [14–25].

Although Wiener introduced path integrals to study stochastic processes, these methods are not commonly used nor familiar to much of the neuroscience or applied mathematics community. There are many textbooks on path integrals but most are geared towards quantum field theory or statistical mechanics [26–28]. Here we give a pedagogical review of these methods specifically applied to SDEs. In particular, we show how to apply the response function method [29, 30], which is particularly convenient to compute desired quantities such as moments.

The main goal of this review is to present methods to compute actual quantities. Thus, mathematical rigor will be dispensed for convenience. This review will be elementary and self-contained. In Sect. 2, we cover moment generating functionals, which expand the definition of generating functions to cover distributions of functions, such as the trajectory of a stochastic process. We continue in Sect. 3 by constructing functional integrals appropriate for the study of SDEs, using the Ornstein–Uhlenbeck process as an example. Section 4 introduces the concept of Feynman diagrams as a tool for carrying out perturbative expansions and introduces the “loop expansion,” a tool for constructing semiclassical approximations. We carry out a perturbative calculation explicitly for the stochastically forced FitzHugh–Nagumo equation in Sect. 5. Finally, Sect. 6 provides the connection between SDEs and equations for the density $p(x,t)$ such as Fokker–Planck equations.

## Moment Generating Functionals

The strategy of path integral methods is to derive a generating function or functional for the moments and response functions for SDEs. The generating functional will be an infinite dimensional generalization for the familiar generating function for a single random variable. In this section we review moment generating functions and show how they can be generalized to functional distributions.

Consider a probability density function (PDF) $P(x)$ for a single real variable *x*. The moments of the PDF are given by

$$ \bigl\langle x^{n} \bigr\rangle = \int x^{n} P(x)\,dx $$

and can be obtained directly by taking derivatives of the generating function

$$ Z(J)= \bigl\langle e^{J x} \bigr\rangle = \int e^{J x} P(x)\,dx, $$

where *J* is a complex parameter, with

$$ \bigl\langle x^{n} \bigr\rangle = \frac{1}{Z(0)} \frac{d^{n}}{d J^{n}}Z(J) \bigg|_{J=0}. $$

Note that in explicitly including $Z(0)$ we are allowing for the possibility that $P(x)$ is not normalized. This freedom will be convenient especially when we apply perturbation theory. The generating function is a Laplace transform of the PDF and is well defined if the PDF is locally integrable.

For example, the generating function for a Gaussian PDF, $P(x)\propto e^{-(x-a)^{2}/(2G)}$, with mean *a* and variance *G*, is

1 $$ Z(J)=\int_{-\infty}^{\infty}e^{-(1/(2G))(x-a)^{2}+J x}\,dx. $$

The integral can be computed by completing the square so that the exponent of the integrand can be written as a perfect square

$$ -\frac{1}{2G}(x-a)^{2}+J x = -A(x-x_{c})^{2}+B. $$

Expanding both sides and equating coefficients yields $x_{c}=JG+a$, $A=1/2G$ and $B=J^{2}G/2+Ja$. The integral in (1) can then be computed to obtain

$$ Z(J)=\int_{-\infty}^{\infty}e^{-(1/2)G^{-1}(x-JG-a)^{2}+J a+J^{2}G/2}\,dx=Z(0)e^{J a+J^{2}G/2}, $$

where

$$ Z(0)=\int_{-\infty}^{\infty}e^{-x^{2}/(2G)}\,dx = \sqrt{2 \pi G} $$

is a normalization factor. The mean of *x* is then given by

$$ \langle x \rangle=\frac{d}{dJ} e^{J a+J^{2}G/2} \bigg|_{J=0}=a. $$

The cumulant generating function is defined as

$$ W(J)=\ln Z(J), $$

so that the cumulants are

$$ \bigl\langle x^{n} \bigr\rangle _{C}= \frac{d^{n}}{dJ^{n}} W(J) \bigg|_{J=0}. $$

In the Gaussian case

$$ W(J)=J a + \frac{1}{2}J^{2}G+\ln Z(0) $$

yielding $\langle x \rangle_{C} =\langle x \rangle= a$ and $\langle x^{2} \rangle _{C}\equiv\operatorname{var}(x)=\langle x^{2} \rangle-\langle x\rangle^{2}= G$, and $\langle x^{n}\rangle_{C}=0$, $n>2$.

The generating function can be generalized to an *n*-dimensional vector $x=\{x_{1},x_{2},\ldots,x_{n}\}$ to become a generating functional that maps the *n*-dimensional vector $J=\{J_{1},J_{2},\dots,J_{n}\}$ to a real number with the form

$$ Z[J]=\int\prod_{l=1}^{n}\, dx_{l} e^{-\sum_{j,k}(1/2) x_{j}G^{-1}_{jk}x_{k}+\sum_{j} J_{j} x_{j}}, $$

where $G^{-1}_{jk}\equiv (G^{-1})_{jk}$ and we use square brackets to denote a functional. This integral can be solved by transforming to orthonormal coordinates, which is always possible if $G^{-1}_{jk}$ is symmetric, as it is for a well-defined probability density. Hence, let $\omega_{\alpha}$ and $v^{\alpha}$ be the *α*th eigenvalue and orthonormal eigenvector of $G^{-1}$, respectively, i.e.

$$ \sum_{j} G^{-1}_{jk} v_{k}^{\alpha}= \omega_{\alpha}v_{j}^{\alpha}$$

and

$$ \sum_{j} v_{j}^{\alpha}v_{j}^{\beta}=\delta_{\alpha\beta}. $$

Now, expand *x* and *J* in terms of the eigenvectors with

$$\begin{aligned} x_{k} =&\sum_{\alpha}c_{\alpha}v^{\alpha}_{k}, \\ J_{k} =&\sum_{\alpha}d_{\alpha}v^{\alpha}_{k}. \end{aligned}$$

Hence

$$ \sum_{j,k} x_{j}G^{-1}_{jk} x_{k}=\sum_{j}\sum _{\alpha,\beta} c_{\alpha}\omega_{\beta}c_{\beta}v_{j}^{\alpha}v_{j}^{\beta}=\sum _{\alpha,\beta} c_{\alpha}\omega_{\beta}c_{\beta}\delta_{\alpha\beta}=\sum_{\alpha}\omega_{\alpha}c^{2}_{\alpha}. $$

Since the Jacobian is 1 for an orthonormal transformation the transformed generating functional in terms of the transformed parameter *d* is

$$\begin{aligned} Z[d] = &\int\prod_{\alpha}dc_{\alpha}e^{\sum_{\alpha}(-(1/2)\omega _{\alpha}c^{2}_{\alpha}+ d_{\alpha}c_{\alpha})} \\ =&\prod_{\alpha}\int_{-\infty}^{\infty}dc_{\alpha}e^{-(1/2)\omega _{\alpha}c^{2}_{\alpha}+ d_{\alpha}c_{\alpha}} \\ =&Z[0]\prod_{\alpha}e^{(1/2)\omega_{\alpha}^{-1} d^{2}_{\alpha}}. \end{aligned}$$

Transforming back yields

$$Z[J]=Z[0]e^{\sum_{jk}1/2J_{j} G_{jk}J_{k} }, $$

where $Z[0]=(2\pi)^{n/2}\sqrt{\det{G}}$.

The moments are given by

$$ \Biggl\langle \prod_{j=1}^{s} x_{j} \Biggr\rangle = \frac {1}{Z[0]}\prod _{j=1}^{s}\frac{\partial}{\partial J_{j}} Z[J] \bigg|_{J_{j}=0}. $$

However, since the exponent is quadratic in the components $J_{l}$, only even powered moments are nonzero. From this we can deduce that

$$ \Biggl\langle \prod_{j=1}^{2s} x_{j} \Biggr\rangle =\sum_{\mathrm{all\ possible\ pairings}} G_{j_{1},j_{2}}\cdots G_{j_{2s-1}j_{2s}}, $$

which is known as Wick’s theorem. Any Gaussian moment about the mean can be obtained by taking the sum of all the possible ways of “contracting” two of the variables. For example,

$$ \langle x_{a} x_{b} x_{c} x_{d} \rangle= G_{ab}G_{cd} +G_{ad}G_{bc}+G_{ac}G_{bd}. $$

In the continuum limit, a generating functional for a function $x(t)$ on the real domain $t\in[0,T]$ is obtained by taking a limit of the generating functional for the vector $x_{j}$. Let the interval $[0,T]$ be divided into *n* segments of length *h* so that $T=nh$ and $x(jh)=x_{j}$, $j\in\{0,1,\dots,n\}$. We then take the limit of $n\rightarrow\infty$ and $h\rightarrow0$ preserving $T= n h$. We similarly identify $J_{j}\rightarrow J(t)$ and $G_{jk}\rightarrow G(s,t)$ and obtain

2 $$\begin{aligned} Z[J] =&\int\mathcal{ D}x(t) e^{- (1/2)\int x(s)G^{-1}(s,t) x(t)\,ds \,dt +\int J(t)x(t)\,dt} \\ =& Z[0]e^{ (1/2)\int J(s)G(s,t)J(t)\,ds \,dt } , \end{aligned}$$

where the measure for integration

$$ \mathcal{ D} x(t)\equiv\lim_{n\rightarrow\infty}\prod _{j=0}^{n} \,dx_{j} $$

is over functions. Although $Z[0]=\lim_{n\rightarrow\infty}(2\pi)^{n/2}(\det G)^{1/2}$ is formally infinite, the moments of the distributional are well defined. The integral is called a path integral or a functional integral. Note that $Z[J]$ refers to a functional that maps different “forms” of the function $J(t)$ over the time domain to a real number. Defining the functional derivative to obey all the rules of the ordinary derivative with

$$\begin{aligned} \frac{\delta J(s)}{\delta J(t)}=\delta(s-t), \end{aligned}$$

the moments again obey

$$ \biggl\langle \prod_{j} x(t_{j}) \biggr\rangle = \frac{1}{Z[0]}\prod_{j} \frac{\delta}{\delta J(t_{j})}Z[J] = \sum_{\mathrm{all\ possible\ pairings}} G(t_{j_{1}},t_{j_{2}}) \cdots G(t_{j_{2s-1}},t_{t_{j_{2s}}}). $$

For example,

$$ \bigl\langle x(t_{1})x(t_{2})\bigr\rangle = \frac{1}{Z[0]}\frac{\delta}{\delta J(t_{1})} \frac{\delta}{\delta J(t_{2})}Z[J]=G(t_{1},t_{2}). $$

We can further generalize the generating functional to describe the probability distribution of a function $\varphi(\vec{x})$ of a real vector $\vec{x}\in R^{n}$, instead of a single variable *t* with

$$\begin{aligned} Z[J] =&\int\mathcal{D}\varphi e^{-\int (1/2) \varphi(\vec {y})G^{-1}(\vec{y},\vec{x}) \varphi(\vec{x}) \,d^{n}y \,d^{n}x +\int J(\vec {x})\varphi(\vec{x})\, d^{n}x} \\ =& Z[0]e^{\int (1/2) J(\vec{y})G(\vec{y},\vec{x})J(\vec{x})\, d^{n}y \,d^{n}x }. \end{aligned}$$

Historically, computing moments and averages of a probability density functional of a function of more than one variable is called field theory. In general, the probability density functional is usually written in exponential form

$$ P[\varphi]=e^{-S[\varphi(\vec{x})]}, $$

where $S[\varphi]$ is called the action and the generating functional is often written as

$$ Z[J]=\int\mathcal{D}\varphi e^{-S[\varphi] + J\cdot\varphi}, $$

where

$$ J\cdot\varphi= \int J(\vec{x})\varphi(\vec{x})\, d^{n}x. $$

For example, the action given by

$$ S[\varphi] = \int\varphi(\vec{x}) G^{-1}\bigl(\vec{x}, \vec{x'}\bigr)\varphi\bigl(\vec {x'}\bigr) \,d^{n}x \,d^{n}x' + g\int\varphi^{4}( \vec{x})\, d^{n}x $$

is called $\varphi^{4}$ (“*φ*-4”) theory.

The analogy between stochastic systems and quantum theory, where path integrals are commonly used, is seen by transforming the time coordinates in the path integrals via $t \rightarrow\sqrt{-1}t$. When the field *φ* is a function of a single variable *t*, then this would be analogous to single particle quantum mechanics where the quantum amplitude can be expressed in terms of a path integral over a configuration variable $\phi(t)$. When the field is a function of two or more variables $\varphi(\vec{r},t)$, then this is analogous to quantum field theory, where the quantum amplitude is expressed as a path integral over the quantum field $\varphi(\vec{r},t)$.

## Application to SDE

Building on the previous section, here we derive a generating functional for SDEs. Consider a Langevin equation,

3 $$ \frac{dx}{dt}=f(x,t)+ g(x,t)\eta(t), $$

on the domain $t\in[0,T]$ with initial condition $x(t_{0})=y$. The stochastic forcing term $\eta(t)$ obeys $\langle\eta(t) \rangle= 0$ and $\langle\eta(t) \eta(t') \rangle= \delta(t - t')$. Equation (3) is to be interpreted as the Ito stochastic differential equation

4 $$ dx=f(x_{t},t)\,dt+ g(x_{t},t)\,dW_{t} , $$

where $W_{t}$ is a Wiener process (i.e. Gaussian white noise), and $x_{t}$ is the value of *x* at time *t*. We show how to generalize to other stochastic processes later. Functions *f* and *g* are assumed to obey all the properties required for an Ito SDE to be well posed [31]. In particular, the stochastic increment $dW_{t}$ does not depend on $f(x_{t},t)$ or $g(x_{t},t)$ (i.e. $x_{t}$ is adapted to the filtration generated by the noise). The choice between Ito and Stratonovich conventions amounts to a choice of the measure for the path integrals, which will be manifested in a condition on the linear response or “propagator” that we introduce below.

The goal is to derive a probability density functional (PDF) and moment generating functional for the stochastic variable $x(t)$. For the path integral formulation, it is more convenient to take $x(t_{0})=0$ in (3) and enforce the initial condition with a source term so that

5 $$ dx=f(x_{t},t)\,dt+ g(x_{t},t)\,dW_{t}+ y\mathbf{1}_{t_{0}}(t) , $$

where $\mathbf{1}_{t_{0}}(t)= 1$ when $t=t_{0}$ (i.e. indicator function). The discretized form of (5) with the Ito interpretation for small time step *h* is given by

6 $$ x_{j+1}-x_{j} = f_{j} h + g_{j} w_{j}\sqrt{h} +y\delta_{j,o} , $$

where $j\in\{0,1,\dots,N\}$, $x_{j}=x(jh+t_{0})$, $T= Nh$, $f_{j}=f(x_{j},jh+t_{0})$, $g_{j}=g(x_{j},jh+t_{0})$, $\delta_{j,k}$ is the Kronecker delta, $x_{0}=0$, and $w_{j}$ is a normally distributed discrete random variable with $\langle w_{j}\rangle=0$ and $\langle w_{j}w_{k}\rangle=\delta_{j,k}$. We use *x* and *w* without indices to denote the vectors $x = (x_{1}, \dots, x_{N})$ and $w = (w_{0}, w_{1}, \dots, w_{N-1})$. Formally, the PDF for the vector *x* conditioned on *w* and *y* can be written as

$$ P[x | w; y]= \prod_{j=0}^{N} \delta \bigl(x_{j+1}-x_{j} - f_{j} h - g_{j} w_{j}\sqrt{h} -y \delta_{j,0}\bigr), $$

i.e. the probability density function is given by the Dirac delta function constrained to the solution of the SDE.

Inserting the Fourier representation of the Dirac delta function,

$$ \delta(z_{j}) =\frac{1}{2\pi} \int e^{-ik_{j} z_{j}}\,dk_{j}, $$

gives

$$ P[x | w;y]=\int\prod_{j=0}^{N} \frac{dk_{j}}{2\pi}e^{-i\sum_{j} k_{j}(x_{j+1}-x_{j}-f_{j} h-g_{j} w_{j}\sqrt{h}-y\delta_{j,0})}. $$

The PDF is now expressed in exponential form.

For zero-mean unit-variance Gaussian white noise, the PDF of $w_{i}$ is given by

$$ P(w_{j})=\frac{1}{\sqrt{2\pi}} e^{-(1/2)w_{j}^{2}}. $$

Hence

$$\begin{aligned} P[x | y] =&\int P[x | w; y] \prod_{j=0}^{N} P(w_{j})\,dw_{j} \\ =&\int\prod_{j=0}^{N} \frac{dk_{j}}{2\pi} e^{-i\sum_{j} k_{j}(x_{j+1}-x_{j}-f_{j} h - y\delta_{j,0})} \\ &{}\times\int\prod_{j=0}^{N} \frac {dw_{j}}{\sqrt{2\pi}} e^{ik_{j}g_{j} w_{j}\sqrt{h}}e^{-(1/2)w_{j}^{2}} \end{aligned}$$

can be integrated by completing the square as demonstrated in the previous section to obtain

$$ P[x | y]=\int\prod_{j=0}^{N} \frac{dk_{j}}{2\pi} e^{-\sum_{j} (ik_{j}) ((x_{j+1}-x_{j})/h-f_{j} -y\delta_{j,0}/h )h+\sum _{j} (1/2) g_{j}^{2} (ik_{j})^{2}h}. $$

In the limit $h\rightarrow0$, $N\rightarrow\infty$ such that $T=Nh$, we can formally use the notation

7 $$ P\bigl[x(t) | y,t_{0}\bigr]=\int\mathcal{ D}\tilde{x}(t) e^{-\int [ \tilde {x}(t) (\dot{x}(t)-f(x(t),t)-y\delta(t-t_{0}) ) - (1/2)\tilde{x}^{2}(t) g^{2}(x(t),t) ]\,dt} $$

with a newly defined complex variable $i k_{j} \rightarrow\tilde{x}(t)$. Although we use a continuum notation for convenience, $x(t)$ need not be differentiable and we interpret the action in terms of the discrete definition. However, all derivatives will be well defined in the perturbative expansions. $P[x(t) | y,t_{0}]$ is a functional of the function $x(t)$ conditioned on two scalars *y* and $t_{0}$. The moment generating functional for $x(t)$ and $\tilde{x}(t)$ is then given by

$$ Z[J,\tilde{J}]=\int\mathcal{D} x(t)\mathcal{D}\tilde{x}(t) e^{-S[x,\tilde{x}] +\int\tilde{J}(t)x(t)\,dt + \int J(t) \tilde{x}(t)\,dt} $$

with action

8 $$ S[x,\tilde{x}]= \int \biggl[ \tilde{x}(t) \bigl(\dot{x}(t)-f\bigl(x(t),t\bigr) -y\delta (t-t_{0})\bigr) - \frac{1}{2}\tilde{x}^{2}(t) g^{2}\bigl(x(t),t\bigr) \biggr]\,dt. $$

The probability density functional can be derived directly from the SDE (3) by taking the path integral over the infinite dimensional Dirac delta functional:

$$\begin{aligned} P\bigl[x(t) | y, t_{0}\bigr] =&\int\mathcal{D}\eta(t) \delta\bigl[ \dot {x}(t)-f(x,t)-g(x,t)\eta(t)-y\delta(t-t_{0})\bigr]e^{-\int\eta^{2}(t)\,dt} \\ =&\int\mathcal{D}\eta(t)\mathcal{D}\tilde{x}(t) e^{-\int\tilde{x}(t) (\dot{x}(t)-f(x,t)-y\delta(t-t_{0})) +\tilde{x}(t)g(x,t)\eta(t)- \eta ^{2}(t)\,dt} \\ =&\int\mathcal{D}\tilde{x}(t) e^{-\int\tilde{x}(t)(\dot {x}(t)-f(x,t)-y\delta(t-t_{0})) +\frac{1}{2}\tilde{x}^{2}(t)g^{2}(x,t)\,dt}, \end{aligned}$$

yielding the action (8).^1^ Owing to the definition $i k_{j} \rightarrow\tilde{x}(t)$ the integrals over $\tilde{x}(t)$ are along the *imaginary* axis, which is why no explicit *i* appears in the action above.

If we perform the $\tilde{x}$ integration in (7) we obtain

$$P\bigl[x(t) | y, t_{0}\bigr] =\mathcal{J} e^{-\int (1/(2 g^{2}(x,t))) [\dot {x}(t)-f(x,t)-y\delta(t-t_{0}) ]^{2}\,dt}, $$

where the Jacobian factor $\mathcal{J}$ depends upon the Ito or Stratonovich convention. For Ito, the Jacobian is 1. This is the Onsager–Machlup formula, which is a useful form for many stochastic applications [2, 13, 28]. For example, there is an intimate connection between the action and the rate function of large deviation theory [13, 32]. However, as we show in the following sections, it is more convenient to not integrate over $\tilde{x}$ for diagrammatic perturbation theory.

In a similar manner, we can define the path integral for more general noise processes than the Wiener process. Let $\eta(t)$ instead be a process with cumulant generating functional $W[J(t)]$ so that the cumulants of $\eta(t)$ (which may depend upon $x(t)$) are given by functional derivatives with respect to $J(t)$. This process will have its own action $S_{\eta}[\eta(t)]$ and the path integral can be written as

$$\begin{aligned} P\bigl[x(t) | y, t_{0}\bigr] =&\int\mathcal{D}\eta(t) \delta\bigl[ \dot{x}(t)-f(x,t)-\eta (t)-y\delta(t-t_{0})\bigr]e^{-S_{\eta}[\eta(t)]} \\ =&\int\mathcal{D}\eta(t)\mathcal{D}\tilde{x}(t) e^{-\int\tilde{x}(t) (\dot{x}(t)-f(x,t)-y\delta(t-t_{0})) +\tilde{x}(t)\eta(t)\,dt -S_{\eta}[\eta(t)]}. \end{aligned}$$

Noting that

$$ \int\mathcal{D}\eta(t)e^{\int\tilde{x}(t)\eta(t)\,dt -S_{\eta}[\eta(t)]} = e^{W[\tilde{x}(t)]} $$

is the definition of the cumulant generating functional for $\eta(t)$, we find that the path integral can be written as

$$ P\bigl[x(t) | y, t_{0}\bigr] = \int\mathcal{D}\eta(t)\mathcal{D} \tilde{x}(t) e^{-\int\tilde{x}(t) (\dot{x}(t)-f(x,t)-y\delta(t-t_{0}))\,dt +W[\tilde{x}(t)]}. $$

In the cases where the input $\eta(t)$ is delta-correlated in time, we obtain

$$ W\bigl[\tilde{x}(t)\bigr] = \sum_{n=1}^{\infty} \int g_{n}\bigl(x(t)\bigr) \tilde{x}(t)^{n} \,dt= \sum _{n=1, m=0}^{\infty}\frac{v_{nm}}{n!} \int \tilde{x}^{n}(t) x^{m}(t)\,dt, $$

where we have Taylor expanded the moments of the noise distribution $g_{n}(x)$. For example, for the Wiener process

$$ W\bigl[\tilde{x}(t)\bigr] = \frac{D}{2} \int\tilde{x}(t)^{2}\,dt, $$

i.e. $v_{20} = D$ and all other $v_{nm} = 0$.

### Ornstein–Uhlenbeck Process

We first demonstrate the path integral approach to computing moments in SDEs for the Ornstein–Uhlenbeck process

$$ \dot{x}(t)+ax(t) -\sqrt{D}\eta(t) =0 $$

with initial condition $x(0)=y$. The action is

$$ S[x,\tilde{x}]=\int \biggl[ \tilde{x}(t) \bigl(\dot{x}(t)+ax(t) - y \delta(t-t_{0}) \bigr) -\frac{D}{2}\tilde{x}^{2}(t) \biggr]\,dt. $$

Defining an inverse propagator

$$ G^{-1}\bigl(t-t'\bigr) = \biggl(\frac{d}{dt} + a \biggr)\delta\bigl(t-t'\bigr) $$

the action can be written as

$$ S[x,\tilde{x}]=\int\tilde{x}(t)G^{-1}\bigl(t-t'\bigr)x \bigl(t'\bigr) \,dt\,dt'-\int y\tilde {x}(t) \delta(t-t_{0})\,dt-\int\frac{D}{2}\tilde{x}(t)^{2}\,dt, $$

and the generating functional is

$$ Z[J,\tilde{J}]=\int\mathcal{D} x(t) \mathcal{D}\tilde{x}(t) e^{-S[x,\tilde{x}] +\int\tilde{J}(t)x(t)\,dt + \int J(t) \tilde{x}(t)\,dt}. $$

This path integral can be evaluated directly as a Gaussian integral since the action is quadratic. However, the generating functional can also be evaluated by expanding the exponent around the “free” action given by $S_{\mathrm{F}}[x(t), \tilde{x}(t)]=\int\tilde{x}(t) G^{-1}(t - t') x(t')\,dt \,dt'$. We will demonstrate this method since it forms the basis for perturbation theory for non-quadratic actions. Expand the integrand of the generating functional as

9 $$\begin{aligned} Z[J,\tilde{J}] =&\int\mathcal{D} x(t)\mathcal{D}\tilde{x}(t) e^{-\int \,dt\,dt' \tilde{x}(t)G^{-1}(t-t')x(t')} \\ &{}\times \biggl(1 +\mu+\frac{1}{2!}\mu ^{2}+\frac{1}{3!} \mu^{3}+\cdots \biggr) , \end{aligned}$$

where

$$ \mu=y\int\tilde{x}(t)\delta(t-t_{0})\,dt+ \int\frac{D}{2} \tilde{x}^{2}(t)\,dt +\int\tilde{J}(t)x(t)\,dt +\int J(t)\tilde{x}(t)\,dt. $$

The generating functional is now expressed as a sum of moments of the free action, which are calculated from the free generating functional

10 $$ Z_{\mathrm{F}}[J,\tilde{J}]=\int\mathcal{D} x(t)\mathcal{D}\tilde{x}(t) e^{-\int \,dt\,dt' \tilde{x}(t)G^{-1}(t-t')x(t') +\int J(t)\tilde{x}(t)\,dt + \int \tilde{J}(t) x(t)\,dt}. $$

Although this integral is similar to (2), there are sufficient differences to warrant an explicit computation. We note again that $\tilde{x}$ is an imaginary variable so this integral corresponds to computing a functional complex Gaussian in two fields. As in Sect. 2, we discretize the time domain with $t\rightarrow t_{k}$, $(x(t_{k}), \tilde{x}(t_{k})) \rightarrow (x_{k}, iy_{k})$, $(J(t_{k}), \tilde{J}(t_{k}))\rightarrow(J_{k}, K_{k}) $,

$$G^{-1}_{kl} = \begin{pmatrix} -1 + a\,dt' & 1 & 0 \cdots\\ 0 & -1 +a\,dt'& 1 \cdots\\ \vdots&\vdots&\ddots \end{pmatrix} $$

and $\int \,dt \rightarrow\sum$. The generating functional is then transformed to

$$\begin{aligned} Z_{\mathrm{F}} =& \int\prod_{m}\,dx_{m} \prod_{k}\frac{dy_{k}}{2\pi} e^{-i \sum_{k} y_{k} (\sum_{l} G_{kl}^{-1} x_{l} - J_{k}) + \sum_{k} K_{k} x_{k}} \\ =&\int\prod_{m}\,dx_{m} \prod _{k} \delta \biggl(\sum_{l} G_{kl}^{-1} x_{l} - J_{k} \biggr)e^{K_{k} x_{k}} \\ =& \frac{1}{\vert \det G_{kl}^{-1}\vert } e^{ \sum_{kl} K_{k}G_{kl} J_{l}}. \end{aligned}$$

In the continuum limit, $dt\rightarrow0$ and $\vert \det G_{kl}^{-1}\vert \rightarrow1$, giving

11 $$ Z_{{\mathrm{F}}}[J,\tilde{J}]=e^{\int\tilde{J}(t)G(t,t'){J}(t')\,dt \,dt'} , $$

where $G(t,t')$ is the operator inverse of $G^{-1}(t,t')$, i.e.

$$ \int \,dt'' G^{-1}\bigl(t,t'' \bigr)G\bigl(t'',t'\bigr) = \biggl( \frac{d}{dt} + a \biggr) G\bigl(t,t'\bigr) = \delta\bigl( t - t'\bigr) . $$

This Green’s function equation can be solved to obtain

$$ G\bigl(t,t'\bigr) = H\bigl(t - t'\bigr) e^{-a(t-t')}, $$

where $H(t)$ is the left continuous Heaviside step function (i.e. $H(0)=0$, $\lim_{t\rightarrow0^{+}} H(t)=1$ and thus $\lim_{t\rightarrow s^{+}} G(t,s)=1$, $G(s,s)=0$). The choice of $H(0)=0$ is consistent with the Ito condition for the SDE and ensures that the configuration variable $x(t)$ is uncorrelated with future values of the stochastic driving term. Other choices for $H(0)$ represent other forms of stochastic calculus (e.g. $H(0) = 1/2$ is the choice consistent with Stratonovich calculus).^2^ Although the generating functional (11) differs from those introduced in Sect. 2 because it is complex and has two source functions *J* and $\tilde{J}$, it still obeys Wick’s theorem.

The free moments are given by

$$ \biggl\langle \prod_{ij} x(t_{i}) \tilde{x}(t_{j}) \biggr\rangle _{\mathrm{F}} =\prod _{ij} \frac{\delta}{\delta\tilde{J}(t_{i})}\frac{\delta}{\delta J(t_{j})}e^{\int\tilde{J}(t)G(t,t')J(t') \,dt\,dt'} \bigg|_{J=\tilde{J}=0} $$

since $Z_{\mathrm{F}}[0,0]=1$. We use a subscript *F* to denote expectation values with respect to the free action. From the action of (11), it is clear the nonzero free moments must have equal numbers of $x(t)$ and $\tilde{x}(t)$ due to Wick’s theorem, which applies here for contractions between $x(t)$ and $\tilde{x}(t)$. For example, one of the fourth moments is given by

$$ \bigl\langle x(t_{1})x(t_{2}) \tilde{x}(t_{3}) \tilde{x}(t_{4})\bigr\rangle _{\mathrm{F}} = G(t_{1},t_{3})G(t_{2},t_{4})+G(t_{1},t_{4})G(t_{2},t_{3}). $$

Now the generating functional for the OU process (9) can be evaluated. The only surviving terms in the expansion will have equal numbers of $x(t)$ and $\tilde{x}(t)$. Thus only terms with factors of $\int\tilde{x}(t_{0})\tilde{J}(t_{1}) x(t_{1})\,dt_{1}$, $(D/2)\int\tilde {x}^{2}(t_{1}) \tilde{J}^{2}(t_{2})x^{2}(t_{2})\,dt_{1}\,dt_{2}$, and $\int\tilde {J}(t_{1})x(t_{1}) J(t_{2})\tilde{x}(t_{2})\,dt_{1}\,dt_{2}$ (and combinations of the three) will survive. For the OU process, the entire series is summable. First consider the case where $D=0$. Because there must be equal numbers of $\tilde{x}(t)$ and $x(t)$ factors in any nonzero moment due to Wick’s theorem, in this case the generating functional has the form

12 $$\begin{aligned} Z =&1 + \sum _{m=1} \frac{1}{m! m!} \\ &{}\times\int \prod _{j,k=1}^{m}\,dt_{j} \,dt_{k} \Biggl\langle \prod_{j,k=1}^{m} \tilde{J}(t_{j})x(t_{j})\tilde{x}(t_{k}) \bigl[y\delta (t_{k}-t_{0}) +J(t_{k}) \bigr] \Biggr\rangle _{\mathrm{F}} . \end{aligned}$$

From Wick’s theorem, the free expectation value in (12) will be a sum over all possible contractions between $x(t)$ and $\tilde {x}(t)$ leading to *m*! combinations. Thus (12) is

$$ Z=\sum_{m=1} \frac{1}{m!} \biggl( y\int \tilde{J}(t_{1})G(t_{1},t_{0})\,dt_{1}+\int \tilde{J}\bigl(t'\bigr) J \bigl(t''\bigr) G\bigl(t',t'' \bigr)\,dt'\,dt'' \biggr)^{m}, $$

which means the series is an exponential function. The other terms in the exponent when $D \ne0$ can be similarly calculated resulting in

13 $$\begin{aligned} Z\bigl[J(t),\tilde{J}(t)\bigr] =& \exp \biggl(y \int\tilde{J}(t_{1})G(t_{1},t_{0})\,dt_{1}+\int\tilde{J}(t_{1}) J(t_{2}) G(t_{1},t_{2})\,dt_{1} \,dt_{2} \\ &{}+\frac{D}{2}\int\tilde{J}(t_{1})\tilde{J}(t_{2}) G \bigl(t_{1},t''\bigr)G \bigl(t_{2},t''\bigr)\,dt''\,dt_{1} \,dt_{2} \biggr). \end{aligned}$$

The cumulant generating functional is

14 $$\begin{aligned} W\bigl[J(t),\tilde{J}(t)\bigr] =&y\int\tilde{J}(t)G(t,t_{0})\,dt+ \int \tilde{J}\bigl(t'\bigr) J\bigl(t''\bigr) G\bigl(t',t''\bigr)\,dt'\,dt'' \\ &{}+\frac{D}{2}\int\tilde{J}\bigl(t'\bigr)\tilde{J} \bigl(t''\bigr) G\bigl(t',t\bigr)G \bigl(t'',t\bigr) \,dt\,dt'\,dt'' . \end{aligned}$$

The only nonzero cumulants are the mean

$$ \bigl\langle x(t) \bigr\rangle = yG(t, t_{0}) , $$

the response function

$$ \bigl\langle x(t_{1}) \tilde{x}(t_{2}) \bigr\rangle _{C} = \frac{\delta }{\delta\tilde{J}(t_{1})}\frac{\delta}{\delta J(t_{2})}W[J,\tilde {J}]_{J=\tilde{J}=0} = G(t_{1},t_{2}) , $$

and the covariance

$$\begin{aligned} \bigl\langle x(t_{1})x(t_{2})\bigr\rangle _{C} \equiv&\bigl\langle x(t_{1})x(t_{2})\bigr\rangle - \bigl\langle x(t_{1})\bigr\rangle \bigl\langle x(t_{2})\bigr\rangle \\ =& \frac{\delta}{\delta\tilde{J}(t_{1})}\frac{\delta}{\delta\tilde {J}(t_{2})}W[J,\tilde{J}]_{J=\tilde{J}=0} \\ =&D\int G(t_{1},t)G(t_{2},t)\,dt. \end{aligned}$$

Closed-form expressions for the cumulants are obtained by using the solution for the propagator *G*. Hence, the mean is

15 $$ \bigl\langle x(t) \bigr\rangle = ye^{-a(t-t_{0})}H(t-t_{0}), $$

the response function is

$$ \bigl\langle x(t_{1}) \tilde{x}(t_{2}) \bigr\rangle = e^{-a(t_{1}-t_{2})}H(t_{1}-t_{2}), $$

and the covariance is

$$ \bigl\langle x(t_{1})x(t_{2})\bigr\rangle _{C}=D \int_{t_{0}}^{t_{2}} e^{-a(t_{1}-t')}e^{-a(t_{2}-t')} H \bigl(t_{1}-t'\bigr)H\bigl(t_{2}-t' \bigr)\,dt'. $$

For $t_{2}\ge t_{1}\ge t_{0}$

$$ \bigl\langle x(t_{1})x(t_{2})\bigr\rangle _{C}=D \frac{e^{2a(t_{1}-t_{2})}-e^{-a(t_{1}+t_{2}-2t_{0})}}{2a}. $$

For $t_{1}=t_{2}=t$

16 $$ \bigl\langle x(t)^{2}\bigr\rangle _{C}=\frac{D}{2a} \bigl(1-e^{-2a(t-t_{0})}\bigr). $$

The generating functional for the OU process could be computed exactly because the SDE could be solved exactly. The advantage of the path integral formulation is that perturbation theory can be applied systematically in the cases where the path integral cannot be completed exactly.

## Perturbative Methods and Feynman Diagrams

If the SDE is nonlinear then the generating functional cannot be computed exactly as in the linear case. However, propagators and moments can be computed perturbatively. The method we use is an infinite dimensional generalization of Laplace’s method for finite dimensional integrals [33]. In fact, the method was used to compute the generating functional for the Ornstein–Uhlenbeck process. The only difference is that for nonlinear SDEs the resulting asymptotic series is not generally summable.

The strategy is again to split the action $S[x,\tilde{x}]= S_{\mathrm{F}} + S_{\mathrm{I}}$, where $S_{\mathrm{F}}$ is called the “free” action and $S_{\mathrm{I}}$ is called the “interacting” action. The generating functional is

17 $$ Z[J,\tilde{J}]=\int\mathcal{D} x\mathcal{D}\tilde{x} e^{-S[x,\tilde {x}] +\int\tilde{J}x \,dt + \int J \tilde{x}\,dt}. $$

The moments satisfy

18 $$ \Biggl\langle \prod_{j}^{m}\prod _{k}^{n}x(t_{j})\tilde{x}(t_{k}) \Biggr\rangle = \frac{1}{Z[0,0]}\prod_{j}^{m} \prod_{k}^{n} \frac{\delta}{\delta\tilde {J}(t_{j})} \frac{\delta}{\delta J(t_{k})} Z \bigg|_{J=\tilde{J}=0}, $$

and the cumulants satisfy

19 $$ \Biggl\langle \prod_{j}^{m}\prod _{k}^{n}x(t_{j})\tilde{x}(t_{k}) \Biggr\rangle _{C} =\prod_{j}^{m} \prod_{k}^{n} \frac{\delta}{\delta\tilde{J}(t_{j})} \frac {\delta}{\delta J(t_{k})} \ln Z \bigg|_{J=\tilde{J}=0}. $$

The generating functional is computed perturbatively by expanding the integrand of (17) around the free action

$$\begin{aligned} Z[J,\tilde{J}] =&\int\mathcal{D} x\mathcal{D}\tilde{x} e^{-S_{\mathrm{F}}[x,\tilde {x}] } \biggl( 1 + S_{\mathrm{I}}+\int\tilde{J}x \,dt + \int J \tilde{x}\,dt \\ &{}+ \frac{1}{2!} \biggl(S_{\mathrm{I}}+\int\tilde{J}x \,dt + \int J \tilde{x}\,dt \biggr)^{2} + \frac{1}{3!} S_{\mathrm{I}}^{3}+ \cdots \biggr). \end{aligned}$$

Hence, the generating functional can be expressed in terms of a series of free moments.

There are two types of expansions depending on whether the nonlinearity is small or the noise source is small. The small nonlinearity expansion is called a weak coupling expansion and the small noise expansion is called a semiclassical, WKB, or loop expansion.

### Weak Coupling Expansion

Consider the example nonlinear SDE

$$ \dot{x}=-ax +bx^{2} + y\delta(t-t_{0}) + \sqrt{D} x^{p/2}\eta(t), $$

where $a >0$, $p\ge0$, and *b* can be of any sign. For example, $p=0$ corresponds to standard additive noise (as in the OU process), while $p=1$ gives multiplicative noise with variance proportional to *x*. The action for this equation is

20 $$\begin{aligned} S[x,\tilde{x}] =& \int \,dt \tilde{x}\bigl(\dot{x}+ax - bx^{2} - y \delta (t-t_{0})\bigr) - \tilde{x}^{2} x^{p} \frac{D}{2} \\ \equiv& S_{\mathrm{F}}[x,\tilde{x}] -y\tilde{x}(t_{0}) - b\int \,dt \tilde {x}(t)x^{2}(t) -\int \,dt \tilde{x}^{2} x^{p} \frac{D}{2} , \end{aligned}$$

where we have defined the free action as $S_{\mathrm{F}}[x,\tilde{x}] = \int \,dt \tilde{x}(\dot{x}+ax)$. We first perform the weak coupling expansion explicitly and then show how the computation can be simplified using diagrammatic methods.

The generating functional for this action is

$$ Z[J,\tilde{J}]=\int\mathcal{D} x\mathcal{D}\tilde{x} e^{-S_{\mathrm{F}}[x,\tilde {x}] + \int \tilde{x} bx^{2}\,dt +\int\tilde{x}y\delta(t-t_{0})\,dt +\int\tilde{x}^{2}x^{n} (D/2)\,dt+\int\tilde{J}x \,dt + \int J \tilde{x}\,dt}. $$

The Taylor expansion of the exponential around the free action gives

$$\begin{aligned} Z[J,\tilde{J}] =&\int\mathcal{D} x\mathcal{D}\tilde{x} e^{-S_{\mathrm{F}}[x,\tilde {x}] } \biggl( 1 + b\int \tilde{x} x^{2}\,dt + \tilde{x}(t_{0})y \\ &{}+ \frac {D}{2}\int\tilde{x}^{2} x^{n} \,dt+\int\tilde{J}x \,dt + \int J \tilde{x}\,dt \\ &{}+ \frac{1}{2!} \biggl(b\int \tilde{x} x^{2}\,dt + \tilde{x}(t_{0})y +\frac {D}{2}\int\tilde{x}^{2} x^{n} \,dt \\ &{}+\int\tilde{J}x \,dt + \int J \tilde{x}\,dt \biggr)^{2} + \cdots \biggr). \end{aligned}$$

Because the free action $S_{\mathrm{F}}$ is bilinear in $\tilde{x}$, *x*, the only surviving terms in the expansion are those with equal numbers of *x* and $\tilde{x}$ factors. Also, because of the Ito condition, $\langle x(t)\tilde{x}(t)\rangle=0$, these pairings must come from *different* terms in the expansion, e.g. the only term surviving from the first line is the very first term, regardless of the value of *p*. All other terms come from the quadratic and higher terms in the expansion. For simplicity in the remainder of this example we limit ourselves to $p=0$. Hence, the expansion includes terms of the form

$$\begin{aligned} Z[J,\tilde{J}] =&\int\mathcal{D} x\mathcal{D}\tilde{x} e^{-S_{\mathrm{F}}[x,\tilde{x}] } \biggl( 1 \\ &{}+ \frac{1}{2!}2 \biggl(b\int\tilde{x}x^{2} \tilde{x}(t_{0})y \,dt +b\int \tilde{x} x^{2}\,dt \int J \tilde{x}\,dt \\ &{}+\int\tilde{J}x \tilde{x}(t_{0}) y \,dt + \int\tilde{J}x \,dt \int J \tilde{x}\,dt \biggr) \\ &{}+ \frac{1}{3!}\frac{3!}{2!}b^{2}\frac{D}{2}\int \tilde{x} x^{2}\,dt \int\tilde {x}x^{2}\,dt\int \tilde{x}^{2}\,dt \\ &{}+ \frac{1}{3!}\frac{3!}{2!}\frac{D}{2}\int \tilde{x}^{2}\,dt \int\tilde {J}x \,dt \int\tilde{J}x \,dt \\ &{} + \frac{1}{3!}3!b\frac{D}{2}\int\tilde{x}x^{2}\,dt \int \tilde{x}^{2}\,dt \int\tilde{J}x \,dt \\ &{}+ \frac{1}{4!}\frac{4!}{2!}b\int\tilde{x}x^{2}\,dt \bigl( \tilde{x}(t_{0}) y\bigr)^{2} \int\tilde{J}x \,dt \\ &{}+ \frac{1}{4!}\frac{4!}{2!2!} \bigl(\tilde{x}(t_{0}) y \bigr)^{2} \int\tilde{J}x \,dt\int\tilde{J}x \,dt \\ &{}+ \frac{1}{5!}5!b\frac{D}{2}\int\tilde{x}x^{2}\,dt \int \tilde{x}^{2}\,dt \tilde{x}(t_{0}) y \int\tilde{J}x \,dt\int \tilde{J}x \,dt+ \cdots \biggr). \end{aligned}$$

Note that this not an exhaustive list of terms up to fifth order. Many of these terms will vanish because of the Ito condition. The combinatorial factors arise from the multiple ways of combining terms in the expansion. There are *n*! ways of combining terms at order *n* and terms with *m* repeats are divided by a factor of *m*!.

Completing the Gaussian integrals using Wick’s theorem then yields

21 $$\begin{aligned} Z[J,\tilde{J}] =& Z_{\mathrm{F}}[0,0]\biggl(1 \\ &{}+ y\int G(t_{1},t_{0})\tilde{J}(t_{1})\,dt_{1} + \int\tilde{J}(t_{1})G(t_{1},t_{2})J (t_{2})\,dt_{1}\,dt_{2} \\ &{}+ D\int G(t_{2},t_{1})G(t_{3},t_{1}) \tilde{J}(t_{2})\tilde{J}(t_{3})\,dt_{1} \,dt_{2} \,dt_{3} \\ &{}+ bD\int G(t_{1},t_{2})^{2}G(t_{3},t_{1}) \tilde{J}(t_{3})\,dt_{1} \,dt_{2} \,dt_{3} \\ &{}+ by^{2}\int G(t_{1},t_{0})^{2} G(t_{2},t_{1}) \tilde{J}(t_{2})\,dt_{1} \,dt_{2} \\ &{}+ y^{2}\int G(t_{1},t_{0}) \tilde{J}(t_{1})\,dt_{1}\int G(t_{2},t_{0}) \tilde {J}(t_{2})\,dt_{2} \\ &{}+ 2bDy\int G(t_{1},t_{2})G(t_{1},t_{0})G(t_{3},t_{1})G(t_{4},t_{2}) \tilde{J}(t_{3})\tilde {J}(t_{4})\,dt_{1}\,dt_{2}\,dt_{3}\,dt_{4} \\ &{}+ \cdots\biggr) , \end{aligned}$$

where the propagator is given by the free action and obeys

$$ \biggl( \frac{d}{dt} + a \biggr) G\bigl(t,t'\bigr) = \delta \bigl( t - t'\bigr) , $$

which is solved by

22 $$ G\bigl(t,t'\bigr) = H\bigl(t - t'\bigr) e^{-a(t-t')} , $$

with $H(0)=0$ as before. We also have $Z_{\mathrm{F}}[0,0]=1$.

The moments and cumulants are obtained from (18) and (19) respectively. For example, the mean is given by

23 $$\begin{aligned} \bigl\langle x(t) \bigr\rangle =& \frac{1}{Z[0,0]}\frac{\delta}{\delta \tilde{J}(t)} Z \bigl[J(t),\tilde{J}(t)\bigr]_{J=0,\tilde{J}=0} \\ =&yG(t,t_{0}) + bD\int G(t,t_{1}) G(t_{1},t_{2})^{2}\,dt_{1} \,dt_{2} \\ &{}+ b y^{2} \int G(t,t_{1}) G(t_{1},t_{0})^{2}\,dt_{1} +\cdots. \end{aligned}$$

The covariance is

$$\begin{aligned} \bigl\langle x(s)x(t) \bigr\rangle =& \frac{\delta}{\delta\tilde{J}(s)} \frac{\delta}{\delta\tilde{J}(t)} Z \bigl[J(t),\tilde{J}(t)\bigr]_{J=0,\tilde {J}=0} \\ =& D\int G(s,t_{1})G(t,t_{1})\,dt_{1}+ y^{2} G(s,t_{0})G(t,t_{0}) \\ &{}+ 2bDy \int G(t_{1},t_{2})G(t_{1},t_{0})G(s,t_{1})G(t,t_{2})\,dt_{1} \,dt_{2} \\ &{}+ 2bDy \int G(t_{1},t_{2})G(t_{1},t_{0})G(t,t_{1})G(s,t_{2})\,dt_{1} \,dt_{2} \cdots. \end{aligned}$$

The first cumulant is the same as the mean but the second cumulant or covariance is

24 $$\begin{aligned} \bigl\langle x(s)x(t) \bigr\rangle _{C} =& \frac{\delta}{\delta\tilde {J}(s)} \frac{\delta}{\delta\tilde{J}(t)} \ln Z\bigl[J(t),\tilde {J}(t)\bigr]_{J=0,\tilde{J}=0} \\ =&\frac{1}{Z} \frac{\delta}{\delta\tilde{J}(s)} \frac{\delta}{\delta\tilde {J}(t)} Z \bigg|_{J=0,\tilde{J}=0}- \frac{\delta}{\delta\tilde {J}(s)} Z\frac{\delta}{\delta\tilde{J}(t)} Z \bigg|_{J=0,\tilde{J}=0} \\ =& D\int G(s,t_{1})G(t,t_{1})\,dt_{1} \\ &{}+ 2bDy \int G(t_{1},t_{2})G(t_{1},t_{0})G(s,t_{1})G(t,t_{2})\,dt_{1} \,dt_{2} \\ &{}+ 2bDy \int G(t_{1},t_{2})G(t_{1},t_{0})G(t,t_{1})G(s,t_{2})\,dt_{1} \,dt_{2} \cdots. \end{aligned}$$

### Diagrammatic Expansion

As can be seen in this example, the terms in the perturbation series become rapidly unwieldy. However, a convenient means to keep track of the terms is to use Feynman diagrams, which are graphs with edges connected by vertices that represents each term in the expansion of a moment. The edges and vertices represent terms (i.e. interactions) in the action and hence SDE, which are combined according to a set of rules that reproduces the perturbation expansion shown above. These are directed graphs (unlike the Feynman diagrams usually used for equilibrium statistical mechanics or particle physics). The flow of each graph, which represents the flow of time, is directed from right to left, points to the left being considered to be at times after points to the right. The vertices represent points in time and separate into two groups: *endpoint* vertices and *interior* vertices. The moment $\langle\prod_{j=1}^{N} x(t_{j}) \prod_{k=1}^{M} \tilde{x}(t_{k}) \rangle$ is represented by diagrams with *Nfinal* endpoint vertices which represent the times $t_{j}$ and *Minitial* endpoint vertices which represent the times $t_{k}$. Interior vertices are determined from terms in the action.

Consider the interacting action expressed as the power series

25 $$ S_{\mathrm{I}} = \sum_{n\ge2,m \ge0} V_{nm} = \sum_{n\ge2,m \ge0} \frac {v^{nm}}{n!}\int _{t_{0}}^{\infty}\,dt \tilde{x}^{n} x^{m} , $$

where *n* and *m* cannot both be ≤1 (those terms are part of the free action). (Nonpolynomial functions in the action are expanded in a Taylor series to obtain this form.) There is a vertex type associated with each $V_{nm}$. The moment $\langle\prod_{j=1}^{N} x(t_{j}) \prod_{k=1}^{M} \tilde{x}(t_{k}) \rangle $ is given by a perturbative expansion of free action moments that are proportional to $\langle\prod_{j=1}^{N} x(t_{j}) \prod_{k=1}^{M} \tilde {x}(t_{k}) V(N_{v}) \rangle_{\mathrm{F}}$ where $V(N_{v})$ represents a product of $N_{v}$ vertices. Each term in this expansion corresponds to a graph with $N_{v}$ interior vertices. We label the *k*th vertex with time $t_{k}$. As indicated in equation (25), there is an integration over each such interior time point, over the interval $(t_{0}, \infty)$. The interaction $V_{nm}$ produces vertices with *n* edges to the left of the vertex (towards increasing time) and *m* edges to the right of the vertex (towards decreasing times). Edges between vertices represent propagators that arise from an application of Wick’s theorem and thus every $\tilde {x}(t')$ must be joined by a factor of $x(t)$*in the future*, i.e. $t>t'$, because $G(t,t') \propto H(t-t')$. Also, since $H(0) = 0$ by the Ito condition, each edge must connect two *different* vertices. All edges must be connected, a vertex for the interaction $V_{nm}$ must connect to *n* edges on the left and *m* edges on the right.

**Fig. 1:**
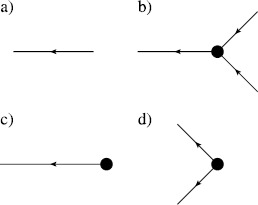
Feynman diagram components for (**a**) an edge, the propagator $G(t,t')$, and vertices (**b**) $\int b\tilde{x} x^{2}\,dt$, (**c**) $\int y \tilde {x} \delta(t-t_{0})\,dt$, and (**d**) $\int\frac{D}{2}\tilde{x}^{2}\,dt$

**Fig. 2:**
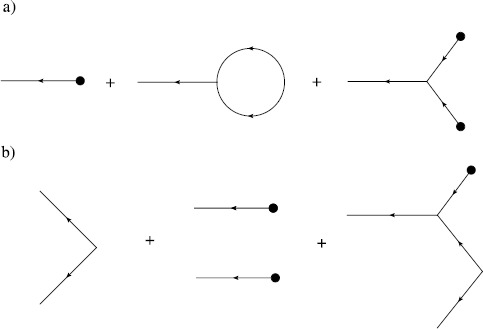
Feynman diagrams for (**a**) the mean and (**b**) second moment for action (20) with $p=0$

Hence, terms at the $N_{v}$th order of the expansion for the moment $\langle\prod_{j=1}^{N} x(t_{j}) \prod_{k=1}^{M} \tilde{x}(t_{k}) \rangle$ are given by directed Feynman graphs with *N* final endpoint vertices, *M* initial endpoint vertices, and $N_{v}$ interior vertices with edges joining all vertices in all possible ways. The sum of the terms associated with these graphs is the value of the moment to $N_{v}$th order. Figure 1 shows the vertices applicable to action (20) with $p=0$. Arrows indicate the flow of time, from right to left. These components are combined into diagrams for the respective moments. Figure 2 shows three diagrams in the sum for the mean and second moment of $x(t)$. The entire expansion for any given moment can be expressed by constructing the Feynman diagrams for each term. Each Feynman diagram represents an integral involving the coefficients of a vertex and propagators. The construction of these integrals from the diagram is encapsulated in the Feynman rules:

(A) For each vertex interaction $V_{nm}$ in the diagram, include a factor of $-\frac{v_{nm}}{n!}$. The minus sign enters because the action appears in the path integral with a minus sign.

(B) If the vertex type, $V_{nm}$ appears *k* times in the diagram, include a factor of $\frac{1}{k!}$.

(C) For each edge between times *t* and $t'$, there is a factor of $G(t,t')$.

(D) For *n* distinct ways of connecting edges to vertices that yield the same diagram, i.e. the same topology, there is an overall factor of *n*. This is the combinatoric factor from the number of different Wick contractions that yield the same diagram.

(E) Integrate over the times *t* of each interior vertex over the domain $(t_{0}, \infty)$.

The diagrammatic expansion is particularly useful if the series can be truncated so that only a few diagrams need to be computed. The weak coupling expansion is straightforward. Suppose one or more of the vertices is associated with a small parameter *α*. Each appearance of that particular vertex diagram contributes a factor of *α* and the expansion can be continued to any order in *α*. The expansion is also generally valid over all time if $G(t,t')$ decays exponentially for large $t-t'$ but can break down if we are near a critical point and $G(t,t')$ obeys a power law. We consider the semiclassical expansion in the next section where the small parameter is the noise strength.

Comparing these rules with the diagrams in Fig. 2, one can see the terms in the expansions in equations (23) and (24), with the exception of the middle diagram in Fig. 2b. An examination of Fig. 2a shows that this middle diagram consists of two copies of the first diagram of the mean. Topologically, the diagrams have two forms. There are connected graphs and disconnected graphs. The disconnected graphs represent terms that can be completely factored into a product of moments of lower order (cf. the middle diagram in Fig. 2b). Cumulants consist only of connected graphs since the products of lower ordered moments are subtracted by definition. The connected diagrams in Fig. 2 lead to the expressions (23) and (24). In the expansion (21), the terms that do not include the source factors *J* and $\tilde{J}$ only contribute to the normalization $Z[0,0]$ and do not affect moments because of (18). In quantum field theory, these terms are called vacuum graphs and consist of closed graphs, i.e. they have no initial or trailing edges. In the cases we consider, all of these terms are 0 if we set $Z[0,0] = 1$.

### Semiclassical Expansion

Recall that the action for the general SDE (3) is

$$ S[x,\tilde{x}]= \int \tilde{x}\bigl(\dot{x}-f\bigl(x(t),t\bigr)\bigr) - \frac{D}{2}\tilde {x}^{2} g^{2}\bigl(x(t),t\bigr)\,dt, $$

where we make explicit a small noise parameter *D*, while *f* and *g* are of order one. Now, rescale the action with the transformation $\tilde{x} \rightarrow\tilde{x}/D$ to obtain

$$\begin{aligned} S[x,\tilde{x}]= \frac{1}{D}\int \tilde{x}\bigl(\dot{x}-f\bigl(x(t),t \bigr)\bigr) - \frac {1}{2}\tilde{x}^{2} g^{2} \bigl(x(t),t\bigr)\,dt. \end{aligned}$$

The generating functional then has the form

26 $$ Z[J, \tilde{J}] = \int\mathcal{D} x(t)\mathcal{D}\tilde{x}(t) e^{-(1/D) (S[x(t), \tilde{x}(t)] - \int\tilde{J}(t)x(t)\,dt - \int J(t) \tilde{x}(t)\,dt ) }. $$

In the limit as $D\rightarrow0$, the integral will be dominated by the critical points of the exponent of the integrand. In quantum theory, these critical points correspond to the “classical” equations of motion (mean field theory in statistical mechanics). Hence, an asymptotic expansion in small *D* corresponds to a semiclassical approximation. In both quantum mechanics and stochastic analysis this is also known as a WKB expansion. According to the Feynman rules for such an action, each diagram gains a factor of *D* for each edge (internal or external) and a factor of $1/D$ for each vertex. Let *E* be the number of external edges, *I* the number of internal edges, and *V* the number of vertices. Then each connected graph now has a factor $D^{I+E-V}$. It can be shown via induction that the number of closed loops *L* in a given connected graph must satisfy $L = I - V + 1$ [26]. To see this, note that for diagrams without loops any two vertices must be connected by at most one internal edge since more than one edge would form a closed loop. Since the diagrams are connected we must have $V = I + 1$ when $L = 0$. Adding an internal edge between any two vertices increases the number of loops by precisely one. Thus we see that the total factor for each diagram may be written $D^{E + L - 1}$. Since the number of external edges is fixed for a given cumulant, the order of the expansion scales with the number of loops.

We can organize the diagrammatic expansion in terms of the number of loops in the graphs. Not surprisingly, the semiclassical expansion is also called the loop expansion. For example, as seen in Fig. 2a the graph for the mean has one external edge and thus to lowest order (graph with no loop), there are no factors of *D*, while one loop corresponds to the order *D* term. The second cumulant or variance has two external edges and thus the lowest order tree level term is order *D* as seen in Fig. 2b. Loop diagrams arise because of nonlinearities in the SDE that couple to moments of the driving noise source. The middle graph in Fig. 2a describes the coupling of the variance to the mean through the nonlinear $x^{2}$ term. This produces a single-loop diagram which is of order *D*, compared to the order 1 “tree” level mean graph. Compare this factor of *D* to that from the tree level diagram for the variance, which is order *D*. This same construction holds for higher nonlinearities and higher moments for general theories. The loop expansion is thus a series organized around the magnitude of the coupling of higher moments to lower moments.

The loop expansion implies that for each order of *D* in the expansion, all diagrams with the same number of loops must be included. In some cases, this could be an infinite number of diagrams. However, one can still write down an expression for the expansion because it is possible to write down the sum of all of these graphs as a set of self-consistent equations. For example, consider the expansion of the mean for action (20) for the case where $D=0$ (i.e. no noise term). The expansion will consist of the sum of all tree level diagrams. From Eq. (23), we see that it begins with

$$ \bigl\langle x(t) \bigr\rangle = yG(t,t_{0}) + b y^{2} \int G(t,t_{1}) G(t_{1},t_{0})^{2}\,dt_{1} +\cdots. $$

In fact, this expansion will be the perturbative expansion for the solution of the ordinary differential equation obtained by discarding the stochastic driving term. Hence, the sum of all tree level diagrams for the mean must satisfy

27 $$ \frac{d}{dt} \bigl\langle x(t) \bigr\rangle _{\mathrm{tree}} = -a \bigl\langle x(t) \bigr\rangle _{\mathrm{tree}}+ b \bigl\langle x(t) \bigr\rangle _{\mathrm{tree}}^{2} +y\delta(t-t_{0}). $$

Similarly, the sum of the tree level diagrams for the linear response, $\langle x(t) \tilde{x}(t') \rangle_{\mathrm{tree}} = G_{\mathrm{tree}}(t,t')$, is the solution of the linearization of (27) around the mean solution with a unit initial condition at $t=t'$, i.e. the propagator equation

28 $$ \frac{d}{dt} G_{\mathrm{tree}}\bigl(t,t'\bigr)= -a G_{\mathrm{tree}}\bigl(t,t'\bigr)+ 2b \bigl\langle x(t) \bigr\rangle _{\mathrm{tree}}G_{\mathrm{tree}}\bigl(t,t'\bigr) + \delta \bigl(t - t'\bigr). $$

**Fig. 3:**
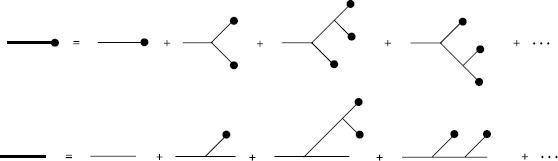
Bold edges represent the sum of all tree level diagrams contributing to that moment. (*Top*) The mean $\langle x(t) \rangle_{\mathrm{tree}}$. (*Bottom*) Linear response $G_{\mathrm{tree}}(t,t')$

The semiclassical approximation amounts to a small noise perturbation around the solution to this equation. We can represent the sum of the tree level diagrams graphically by using bold edges, which we call “classical” edges, as in Fig. 3. We can then use the classical edges within the loop expansion to compute semiclassical approximations to the moments of the solution to the SDE.

Consider the case of $p=0$ in action (20). The one-loop semiclassical approximation of the mean is given by the sum of the first two graphs in Fig. 2a with the thin edges replaced by bold edges. For the covariance, the first graph in Fig. 2b suffices, again with thin edges replaced by bold edges. These graphs are equivalent to the equations:

29 $$ \bigl\langle x(t) \bigr\rangle = \bigl\langle x(t) \bigr\rangle _{\mathrm{tree}} + b D\int_{t_{0}}^{t}\,dt_{1} \int _{t_{0}}^{t_{1}}\,dt_{2} G_{\mathrm{tree}}(t,t_{2}) G_{\mathrm{tree}}(t_{2},t_{1})^{2} $$

and

30 $$ \bigl\langle x(t) x\bigl(t'\bigr) \bigr\rangle _{C} = D \int_{t_{0}}^{\min(t,t')}\,dt_{1} G_{\mathrm{tree}}(t, t_{1}) G_{\mathrm{tree}}\bigl(t',t_{1} \bigr). $$

Using (30) in (29) gives

$$ \bigl\langle x(t) \bigr\rangle = \bigl\langle x(t) \bigr\rangle _{\mathrm{tree}} + b D \int_{t_{0}}^{t}\,dt_{1} G_{\mathrm{tree}}(t,t_{2}) \bigl\langle x(t_{2}) x(t_{2}) \bigr\rangle _{C}. $$

This approximation is first order in *D* for the mean (one loop) and covariance (tree level).

**Fig. 4:**
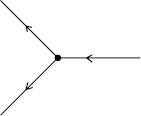
Vertex for multiplicative noise with $p=1$ in the action (20). This vertex replaces the one in Fig. 1d

**Fig. 5:**
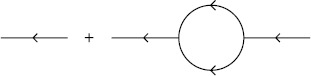
Feynman diagrams for the linear response, $\langle x(t) \tilde {x}(t') \rangle$, to one-loop order

Now consider the one-loop corrections when $p=1$ in action (20). First consider the linear response, $\langle x(t) \tilde {x}(t') \rangle$. For simplicity, we will assume the initial condition $y=0$. In this case, the vertex in Fig. 1d now appears as in Fig. 4. The linear response $\langle x(t) \tilde{x}(t') \rangle$ will be given by the sum of all diagrams with one entering edge and one exiting edge. At tree level, there is only one such graph, equal to $G(t,t')$, given in (22). At one-loop order, we can combine the vertices in Figs. 1b and 1d to get the second graph shown in Fig. 5 to obtain

$$\begin{aligned} \bigl\langle x(t) \tilde{x}\bigl(t'\bigr) \bigr\rangle =& G \bigl(t,t'\bigr) + bD \int \,dt_{1} \,dt_{2} G(t,t_{2}) G(t_{2}, t_{1})^{2}G \bigl(t_{2}, t'\bigr) \\ =& e^{-a(t-t')}H\bigl(t - t'\bigr) \biggl[ 1+ bD \biggl( \frac{t - t'}{a} + \frac {1}{a^{2}} \bigl(e^{-a(t-t')} - 1 \bigr) \biggr) \biggr]. \end{aligned}$$

This loop correction arises because of two types of vertices. There are vertices that we call “branching” (as in Fig. 4), which have more exiting edges then entering edges. The opposite case occurs for those vertices which we call “aggregating.” Noise terms in the SDE produce branching vertices. As can be seen from the structure of the Feynman diagrams, all moments can be computed exactly when the deterministic part of the SDE is linear because it only involves convolving the propagator (i.e. Green’s function) of the deterministic part of the SDE with the driving noise term, as in the case of the OU process above. On the other hand, nonlinearities give rise to aggregating vertices.

Combining the diagrams in Figs. 1 and 4, the analog to Eqs. (29) and (30) for $p=1$ are

31 $$ \bigl\langle x(t) \bigr\rangle = \bigl\langle x(t) \bigr\rangle _{\mathrm{tree}} + b D \int_{t_{0}}^{t}\,dt_{1} \int _{t_{0}}^{t_{1}}\,dt_{2} G_{\mathrm{tree}}(t,t_{2}) G_{\mathrm{tree}}(t_{2},t_{1})^{2} \bigl\langle x(t) \bigr\rangle _{\mathrm{tree}} $$

and

32 $$ \bigl\langle x(t) x\bigl(t'\bigr) \bigr\rangle _{C} = D \int_{t_{0}}^{\min(t,t')}\,dt_{1} G_{\mathrm{tree}}(t, t_{1}) G_{\mathrm{tree}}\bigl(t',t_{1} \bigr)\bigl\langle x(t) \bigr\rangle _{\mathrm{tree}} . $$

Using the definition of $G_{\mathrm{tree}}(t,t')$, the self-consistent semiclassical approximation for $\langle x(t) \rangle$ to one-loop order is

$$ \frac{d}{dt}\bigl\langle x(t) \bigr\rangle + a\bigl\langle x(t) \bigr\rangle - b \bigl\langle x(t) \bigr\rangle ^{2} = b D \int _{t_{0}}^{t}\,dt_{1} G_{\mathrm{tree}}(t,t_{1})^{2} \bigl\langle x(t) \bigr\rangle $$

or

$$ \frac{d}{dt}\bigl\langle x(t) \bigr\rangle + a\bigl\langle x(t) \bigr\rangle - b \bigl\langle x(t) \bigr\rangle ^{2} = b \int _{t_{0}}^{t}\,dt_{1} \bigl\langle x(t_{1}) x(t_{1}) \bigr\rangle _{C}. $$

The semiclassical approximation known as the “linear noise” approximation takes the tree level computation for the mean and covariance. The formal way of deriving these self-consistent equations is via the *effective action*, which is beyond the scope of this review. We refer the interested reader to [26].

An alternative way of deriving the loop expansion equations is to examine deviations away from the mean directly by transforming to a new variable $z=x-\bar{x}$ where $\bar{x}\stackrel{\mathrm{def}}{=} \langle x(t) \rangle_{\mathrm{tree}}$ satisfies the SDE with zero noise (27). The action then becomes

33 $$ S[x,\tilde{x}] = \int dt \tilde{x}\bigl(\dot{z}+az -2\bar{x}z- bz^{2} \bigr) - \tilde{x}^{2} (z+\bar{x})^{p}\frac{D}{2} . $$

The propagator for this action is now given immediately by (28). At tree level, $\langle z \rangle_{\mathrm{tree}}=0$ by definition. At order *D*, the mean is given by the second diagram in Fig. 2a, which immediately gives (29) and (31). Likewise, the variance will be given by the diagram in Fig. 1d leading to (30) and (32).

## FitzHugh–Nagumo Model

These methods can also be applied to higher dimensions. As an example, consider the noise-driven FitzHugh–Nagumo neuron model:

34 $$\begin{aligned} \dot{v} =& v-\frac{1}{3}v^{3}- w + I + v_{0} \delta(t-t_{0}) +\sqrt{D}\eta (t), \end{aligned}$$

35 $$\begin{aligned} \dot{w} =& c(v+a - bw ) +w_{0}\delta(t-t_{0}), \end{aligned}$$

where *v* is the neuron potential, *w* is a recovery variable, *a*, *b*, and *c* are positive parameters, *D* is the noise amplitude, and initial conditions are $v_{0}$, $w_{0}$. We will consider a semiclassical or WKB expansion in terms of *D*. We wish to compute the means, variances, and covariance for *v* and *w* as a loop expansion in *D*.

We first must formally construct a joint PDF for variables *v* and *w*. As both equations in the system must be satisfied simultaneously, we write

$$\begin{aligned} P\bigl[x(t) | y, t_{0}\bigr] =&\int \mathcal{D}\eta(t) \delta \biggl[ \dot{v}-v+\frac {1}{3}v^{3}+ w - I-\sqrt{D}\eta(t)-v_{0} \delta(t-t_{0}) \biggr] \\ &{} \times\delta \bigl[\dot{w}-c (v + a - b w)-w_{0} \delta(t-t_{0}) \bigr]e^{-S_{\eta}[\eta(t)]}, \end{aligned}$$

which leads to the action

$$\begin{aligned} S =&\int \biggl[\tilde{v} \biggl(\dot{v}-v+\frac{1}{3}v^{3}+ w - I-\sqrt {D}\eta(t)-v_{0}\delta(t-t_{0}) \biggr)- \frac{D}{2}\tilde{v}^{2} \\ &{} + \tilde{w} \bigl(\dot{w}-c (v + a - b w)-w_{0} \delta(t-t_{0}) \bigr) \biggr]\,dt. \end{aligned}$$

We now transform to deviations around the mean with $\nu= v-V$ and $\omega= w -W$, with $V\stackrel{\mathrm{def}}{=}\langle v\rangle_{\mathrm{tree}}$ and $W\stackrel{\mathrm{def}}{=}\langle w \rangle_{\mathrm{tree}}$, where

36 $$\begin{aligned} \dot{V} =& V-\frac{1}{3}V^{3}- W + I , \end{aligned}$$

37 $$\begin{aligned} \dot{W} =& c(V+a - bW ). \end{aligned}$$

The transformed action is

$$ S=\int \biggl[\tilde{v} \biggl(\dot{\nu}-\nu+ V^{2}\nu+V \nu^{2}+\frac {1}{3}\nu^{3} + \omega \biggr)- \frac{D}{2}\tilde{v}^{2} + \tilde{w} \bigl(\dot{\omega}-c (\nu- b \omega) \bigr) \biggr]\,dt $$

which we can rewrite as

38 $$ S=\int \biggl[\int \tilde{\psi}^{T}(t)\cdot\mathbf{G}^{-1} \bigl(t,t'\bigr)\cdot\psi \bigl(t'\bigr)\,dt'+\tilde{v}V\nu^{2}+\frac{1}{3}\tilde{v} \nu^{3} -\frac{D}{2}\tilde {v}^{2} \biggr]\,dt, $$

where

$$\begin{aligned} \psi =& \begin{pmatrix} \nu\\ \omega \end{pmatrix} , \quad\quad \tilde{\psi}= \begin{pmatrix} \tilde{v}\\ \tilde{w} \end{pmatrix} ,\\ \mathbf{G}^{-1}\bigl(t,t'\bigr) =& \begin{pmatrix} (\frac{d}{dt} +V^{2} - 1 ) \delta(t-t') & \delta(t-t')\\ -c\delta(t-t')& (\frac{d}{dt}+c b )\delta(t-t') \end{pmatrix} . \end{aligned}$$

The propagator

$$ \mathbf{G }\bigl(t,t'\bigr)= \begin{pmatrix} G^{v}_{\nu}(t,t') & G^{w}_{\nu}(t,t')\\ G^{v}_{\omega}(t,t') & G^{w}_{\omega}(t,t') \end{pmatrix} $$

satisfies

$$ \int\mathbf{G}^{-1}\bigl(t,t''\bigr)\mathbf{G} \bigl(t'',t'\bigr)\,dt'' = \begin{pmatrix} \delta(t-t') & 0\\ 0 & \delta(t-t') \end{pmatrix} $$

or

39 $$\begin{aligned} \biggl(\frac{d}{dt} +V^{2} - 1 \biggr)G^{v}_{\nu}+ G^{v}_{\omega} =& \delta \bigl(t-t' \bigr), \end{aligned}$$

40 $$\begin{aligned} \biggl(\frac{d}{dt}+c b \biggr)G^{v}_{\omega}- c G^{v}_{\nu} =&0, \end{aligned}$$

41 $$\begin{aligned} \biggl(\frac{d}{dt} +V^{2} - 1 \biggr)G^{w}_{\nu}+ G^{w}_{\omega} =& 0, \end{aligned}$$

42 $$\begin{aligned} \biggl(\frac{d}{dt}+c b \biggr)G^{w}_{\omega}- c G^{w}_{\nu} =&\delta\bigl(t-t' \bigr). \end{aligned}$$

**Fig. 6:**
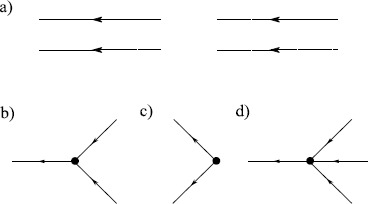
Vertices and propagators for Feynman diagrams in the Fitzhugh–Nagumo model. (**a**) Propagators $G_{\nu}^{v}$ (*solid–solid*), $G\nu ^{w}$ (*solid–dashed*), $G_{\omega}^{v}$ (*dashed–solid*), $G_{\omega}^{w}$ (*dashed–dashed*), (**b**) vertex $\int \tilde{v}V\nu^{2}\,dt$, (**c**) vertex $\int \frac{D}{2} \tilde{v}^{2}\,dt$, (**d**) vertex $\int\frac{1}{3} \tilde{v}\nu^{2}\,dt$

The Feynman diagrams for the four propagators (39)–(42) and the two vertices in the action (38) are shown in Fig. 6.

**Fig. 7:**
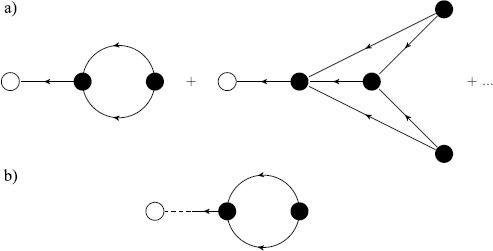
(**a**) Diagrammatic expansion for the mean of $v(t)$, showing the one-loop and an example two-loop graph. (**b**) Diagrams for $w(t)$ are topologically identical with the replacement of the leftmost line by the $G^{v}_{\omega}(t,t')$ propagator

The mean of *v* is $\langle v \rangle= V + \langle\nu\rangle$ and the mean of *w* is $\langle w \rangle= W+ \langle\omega\rangle$. The diagrams for $\langle\nu\rangle$, and $\langle\omega\rangle$ are shown in Fig. 7. $\langle\nu\rangle$ is given by joining the two vertex diagrams in Figs. 6b and 6c to obtain Fig. 7a, which is topologically equivalent to the middle diagram in Fig. 2a:

43 $$ \bigl\langle \nu(t)\bigr\rangle _{t_{0}} =-D \int_{t_{0}}^{t} \,dt_{1}\int_{t_{0}}^{t} \,dt_{2} V(t)G_{\nu}^{v}(t,t_{2}) G_{\nu}^{v}(t_{2},t_{1}) G_{\nu}^{v}(t_{2},t_{1}), $$

where the subscript indicates that this is an ensemble average of $\nu (t)$ in the domain $[t_{0},t]$. $\langle\omega\rangle$ is given by the same diagram as $\langle\nu\rangle$ except that propagator $G_{\omega}^{v}(t,t_{2})$ replaces $G_{\nu}^{v}(t,t_{2})$:

44 $$ \bigl\langle \omega(t)\bigr\rangle _{t_{0}}=-D \int_{t_{0}}^{t} \,dt_{1}\int_{t_{0}}^{t} \,dt_{2} V(t)G_{\omega}^{v}(t,t_{2}) G_{\nu}^{v}(t_{2},t_{1}) G_{\nu}^{v}(t_{2},t_{1}). $$

**Fig. 8:**
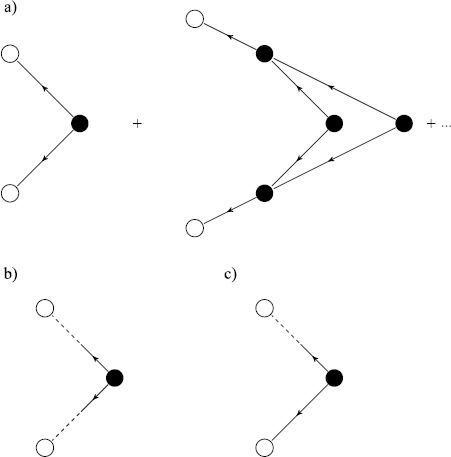
(**a**) Diagrammatic expansion for the two-point correlation $\langle v(t) v(t') \rangle$, showing the tree level and an example one-loop graph. Diagrams for (**b**) $\langle w(t) w(t') \rangle$ and (**c**) $\langle v(t) w(t') \rangle$ are topologically identical with the replacement of the appropriate external lines by the $G^{v}_{\omega}(t,t')$ propagator

The diagrams for the variances and covariances (two-point cumulants) are shown in Fig. 8 The variance of *v* is $\langle\nu(t)\nu(t')\rangle_{C}$ in Fig. 8a is found by using Fig. 6c adjoined to two $G_{\nu}^{v}$ propagators:

45 $$ C_{\nu\nu}\bigl(t,t';t_{0}\bigr) \stackrel{ \mathrm{def}}{=}\bigl\langle \nu(t)\nu\bigl(t'\bigr)\bigr\rangle _{C} = D\int_{t_{0}}^{\min(t,t')}\,dt_{1} G_{\nu}^{v}(t,t_{1})G_{\nu}^{v} \bigl(t',t_{1}\bigr) . $$

The variance of *w* is $\langle\omega(t)\omega(t')\rangle_{C}$ in Fig. 8b is also given by Fig. 6c but adjoined to two $G_{\omega}^{v}$ propagators:

46 $$ C_{\omega\omega}\bigl(t,t';t_{0}\bigr) \stackrel{ \mathrm{def}}{=} \bigl\langle \omega(t)\omega \bigl(t'\bigr)\bigr\rangle _{C} = D\int_{t_{0}}^{\min(t,t')}\,dt_{1} G_{\omega}^{v}(t,t_{1})G_{\omega}^{v} \bigl(t',t_{1}\bigr) . $$

Finally, the covariance of *v* and *w* is $\langle\nu(t)\omega (t')\rangle_{C}$ in Fig. 8c is given by Fig. 6c adjoined to the $G_{\nu}^{v}$ and $G_{\omega}^{v}$ propagators:

47 $$ C_{\nu\omega}\bigl(t,t';t_{0}\bigr) \stackrel{ \mathrm{def}}{=} \bigl\langle \nu(t)\omega \bigl(t'\bigr)\bigr\rangle _{C} = D\int_{t_{0}}^{\min(t,t')}\,dt_{1} G_{\nu}^{v}(t,t_{1})G_{\omega}^{v}\bigl(t',t_{1}\bigr) . $$

To evaluate these expressions we first solve the deterministic equations (37) to obtain $V(t)$ and $W(t)$. We then use $V(t)$ in (39)–(42) and solve for the four propagators, which go into the expressions for the moments. When the solutions of (37) are fixed points, then we can find closed-form solutions for all the equations. Otherwise, we may need to solve some of the equations numerically. However, instead of having to average over many samples of the noise distribution, we only need to solve a small set of equations once to obtain the moments.

Here we give the example solution for the fixed point given by the solution of

48 $$\begin{aligned} 0 =& V-\frac{1}{3}V^{3}- W + I , \end{aligned}$$

49 $$\begin{aligned} 0 =& c(V+a - bW ). \end{aligned}$$

The propagator equations are pairwise coupled and thus can easily be solved by Laplace transforms or any other means to obtain

50 $$\begin{aligned} G^{v}_{\nu}\bigl(t,t'\bigr) =&e^{-r(t-t')} \biggl( \cos\bigl(\varOmega\bigl(t-t'\bigr)\bigr) - \frac {r-bc}{\varOmega}\sin\bigl(\varOmega\bigl(t-t'\bigr)\bigr) \biggr)H \bigl(t-t'\bigr), \end{aligned}$$

51 $$\begin{aligned} G^{v}_{\omega}\bigl(t,t'\bigr) =& \frac{c}{\varOmega}e^{-r(t-t')}\sin\bigl(\varOmega \bigl(t-t'\bigr) \bigr)H\bigl(t-t'\bigr), \end{aligned}$$

52 $$\begin{aligned} G^{w}_{\nu}\bigl(t,t'\bigr) =& \frac{1}{\varOmega}e^{-r(t-t')}\sin\bigl(\varOmega \bigl(t-t'\bigr) \bigr)H\bigl(t-t'\bigr), \end{aligned}$$

53 $$\begin{aligned} G^{w}_{\omega}\bigl(t,t'\bigr) =&-e^{-r(t-t')} \biggl( \cos\bigl(\varOmega\bigl(t-t'\bigr)\bigr) + \frac {r-bc}{\varOmega}\sin\bigl(\varOmega\bigl(t-t'\bigr)\bigr) \biggr)H \bigl(t-t'\bigr), \end{aligned}$$

where $r=(1/2)(V^{2}-1+b c)$,

$$\varOmega=(1/2)\sqrt{-V^{4}+2(1+b c) V^{2} - b^{2} c^{2} -2(b-2)c -1}. $$

We can now insert these propagators into the variance and covariance equations:

54 $$\begin{aligned} \bigl\langle \nu(t)\bigr\rangle _{t_{0}} =&-DV \int_{t_{0}}^{t} \,dt_{2} e^{-r(t-t_{2})} \biggl( \cos\bigl(\varOmega(t-t_{2}) \bigr) - \frac{r-2bc}{2\varOmega}\sin\bigl(\varOmega(t-t_{2})\bigr) \biggr) \\ &{}\times\int_{t_{0}}^{t_{2}}\,dt_{1} e^{-2r(t_{2}-t_{1})} \\ &{}\times \biggl( \cos\bigl(\varOmega (t_{2}-t_{1})\bigr) - \frac{r-bc}{\varOmega}\sin\bigl(\varOmega(t_{2}-t_{1})\bigr) \biggr)^{2}, \end{aligned}$$

55 $$\begin{aligned} \bigl\langle \omega(t)\bigr\rangle _{t_{0}} =&-DV\frac{c}{\varOmega} \int _{t_{0}}^{t} \,dt_{2} e^{-r(t-t_{2})}\sin \bigl(\varOmega(t-t_{2})\bigr) \\ &{}\times\int_{t_{0}}^{t_{2}}\,dt_{1} e^{-2r(t_{2}-t_{1})} \\ &{}\times \biggl( \cos\bigl(\varOmega (t_{2}-t_{1})\bigr) - \frac{r-bc}{\varOmega}\sin\bigl(\varOmega(t_{2}-t_{1})\bigr) \biggr)^{2}, \end{aligned}$$

56 $$\begin{aligned} & \begin{aligned} C_{\nu\nu}\bigl(t,t';t_{0}\bigr) =& D\int _{t_{0}}^{\min(t,t')}\,dt_{1} e^{-r(t-t_{1})} \\ & \ \times \biggl( \cos\bigl(\varOmega(t-t_{1})\bigr) - \frac{r-2bc}{2\varOmega}\sin \bigl(\varOmega(t-t_{1})\bigr) \biggr) \end{aligned} \end{aligned}$$

57 $$\begin{aligned} &{}\times e^{-r(t-t_{1})} \biggl( \cos\bigl(\varOmega\bigl(t'-t_{1} \bigr)\bigr) - \frac {r-2bc}{2\varOmega}\sin\bigl(\varOmega\bigl(t'-t_{1} \bigr)\bigr) \biggr), \end{aligned}$$

58 $$\begin{aligned} C_{\omega\omega}\bigl(t,t';t_{0}\bigr) =& D \frac{c^{2}}{\varOmega^{2}}\int_{t_{0}}^{\min (t,t')}\,dt_{1} e^{-r(t-t_{1})} \\ &{}\times\sin\bigl(\varOmega(t-t_{1})\bigr)e^{-r(t'-t_{1})}\sin \bigl(\varOmega\bigl(t'-t_{1}\bigr)\bigr), \end{aligned}$$

59 $$\begin{aligned} & \begin{aligned} C_{\nu\omega}\bigl(t,t';t_{0}\bigr) = &D \frac{c}{\varOmega}\int_{t_{0}}^{\min(t,t')}\,dt_{1} e^{-r(t-t_{1})} \\ &\ \times\biggl( \cos\bigl(\varOmega(t-t_{1})\bigr) - \frac{r-2bc}{2\varOmega }\sin\bigl(\varOmega(t-t_{1})\bigr) \biggr) \end{aligned} \end{aligned}$$

60 $$\begin{aligned} &{}\times e^{-r(t-t_{1})}\sin\bigl(\varOmega(t-t_{1})\bigr) . \end{aligned}$$

**Fig. 9:**
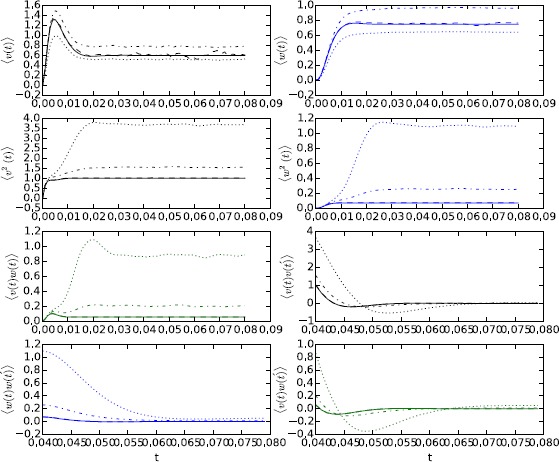
Comparison of ensemble average simulations vs. analytic expansions for the Fitzhugh–Nagumo model. For each moment, we show the one-loop (for the mean) or tree (for the two-point cumulants) level approximation (*solid line*) along with simulations for $D = 0.001$ (*dashed line*), $D = 0.01$ (*dot–dashed* line), and $D=0.015$ (*dotted line*). Means have been scaled by *DV* and two-point cumulants have been scaled by *D* so they can be compared directly

**Fig. 10:**
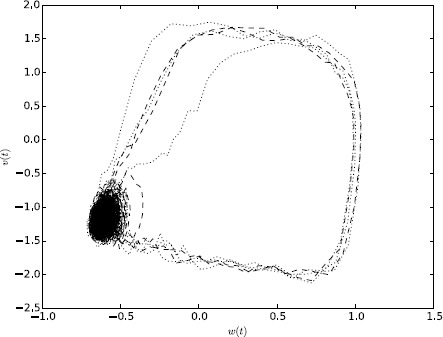
Phase plane portrait for trajectories in the Fitzhugh–Nagumo model for $D = 0.01$ (*solid line*), $D = 0.015$ (*dashed*), and $D=0.02$ (*dotted*). For $D = (0.015, 0.02)$, one can see the large excursions away from the fixed point which appear as the bifurcation becomes closer. These excursions contribute to the ensemble averages and thus the divergence in the diagrammatic series

These expressions not only capture the stationary values but also transient effects. The integrals can all be performed in closed form and result in long expressions of exponential and trigonometric functions, which we do not include here. Figure 9 shows the comparison between these perturbative estimates and numerical simulations for three different noise values, $D=0.001, 0.01, 0.015$. We used the parameters $a=0.7$, $b=0.8$, $c=0.1$, $I=0$, which give rise to fixed points $V=-1.1994$ and $W=-0.62426$ and propagator parameters $r=0.25928$ and $\varOmega=0.26050$. Numerical simulations are averages over one million samples. The estimates fit the data extremely well for $D=0.001$ but start to break down for $D=0.01$. The reason is that the perturbation expansion is only valid in the vicinity of the fixed point but as the noise strength increases the probability to escape from the fixed point and enter an excitable orbit becomes significant. Phase plane portraits in Fig. 10 show that for $D=0.001$ the orbit stays near the stable fixed point but for the larger noise values, there are large excursions away from the fixed point when the orbit crosses an “escape” threshold. The probability to escape can also be estimated using path integrals and WKB theory as detailed in [13, 25]. Software for performing simulations and integrals is available upon request.

## Connection to Fokker–Planck Equation

In stochastic systems, one is often interested in the PDF $p(x,t)$, which gives the probability density of position *x* at time *t*. This is in contrast with the probability density functional $P[x(t)]$ which is the probability density of all possible functions or paths $x(t)$. Previous sections have been devoted to computing the moments of $P[x(t)]$, which provide the moments of $p(x,t)$ as well. In this section we leverage knowledge of the moments of $p(x,t)$ to determine an equation it must satisfy. In simple cases, this equation is a Fokker–Planck equation for $p(x,t)$.

The PDF $p(x,t)$ can be formally obtained from $P[x(t)]$ by marginalizing over the interior points of the function $x(t)$. Consider the transition probability $U(x_{1},t_{1} | x_{0}, t_{0})$ between two points $x_{0}$, $t_{0}$ and $x_{1}$, $t_{1}$. This is equal to $p(x,t)$ given the initial condition $p(x,t_{0}) = \delta(x - x_{0})$. In terms of path integrals this can be expressed as

$$ U(x_{1},t_{1}|x_{0},t_{0}) = \int^{(x(t_{1})=x_{1})}_{(x(t_{0})=x_{0})} \mathcal{D}x(t) P\bigl[x(t) \bigr], $$

where the upper limit in the integral is fixed at $x(t_{1})=x_{1}$ and the lower at $x(t_{0})=x_{0}$. The lower limit appears as the initial condition term in the action and can thus be considered part of $P[x(t)]$. The upper limit on the path integral can be imposed with a functional Dirac delta via

$$ U(x_{1},t_{1}|x_{0},t_{0}) = \int\mathcal{D}x(t) \delta\bigl(x(t_{1})-x_{1}\bigr)P \bigl[x(t)\bigr], $$

which in the Fourier representation is given by

$$ U(x_{1},t_{1}|x_{0},t_{0}) = \frac{1}{2\pi i}\int dJ\int\mathcal{D}x(t) e^{J (x(t_{1})-x_{1})}P\bigl[x(t)\bigr], $$

where the contour for the *J* integral runs along the imaginary axis. This can be rewritten as

61 $$ U(x_{1},t_{1}|x_{0},t_{0}) = \frac{1}{2\pi i}\int dJ e^{-J(x_{1}-x_{0})} Z_{\mathrm{CM}}(J) $$

in terms of an initial condition centered moment generating function

$$ Z_{\mathrm{CM}}(J) = \int\mathcal{D}x e^{J (x(t_{1})-x_{0})}P\bigl[x(t)\bigr], $$

where the measure $\mathcal{D}x(t)$ is defined such that $Z_{\mathrm{CM}}(0)=1$. Note that this generating function $Z_{\mathrm{CM}}(J)$ is different from the generating functionals we presented in previous sections. $Z_{\mathrm{CM}}(J)$ generates moments of the deviations of $x(t)$ from the initial value $x_{0}$ at a specific point in time *t*. Taylor expanding the exponential gives

$$ Z_{\mathrm{CM}}(J) = 1+\sum_{n=1}^{\infty}\frac{1}{n!}J^{n} \bigl\langle \bigl(x(t_{1})-x_{0} \bigr)^{n}\bigr\rangle _{x(t_{0})=x_{0}}, $$

where

$$ \bigl\langle \bigl(x(t_{1})-x_{0}\bigr)^{n} \bigr\rangle _{x(t_{0})=x_{0}} = \int\mathcal{D}x \bigl(x(t_{1})-x_{0} \bigr)^{n}P\bigl[x(t)\bigr] . $$

Inserting into (61) gives

$$ U(x_{1},t_{1}|x_{0},t_{0}) = \frac{1}{2\pi i }\int dJ e^{-J(x_{1}-x_{0})} \Biggl(1+\sum _{n=1}^{\infty}\frac{1}{n!}J^{n} \bigl\langle \bigl(x(t_{1})-x_{0}\bigr)^{n}\bigr\rangle \Biggr). $$

Using the identity

$$ \frac{1}{2\pi i}\int dJ e^{-J(x_{1}-x_{0})} J^{n} = \biggl(- \frac{\partial }{\partial x_{1}} \biggr)^{n}\delta(x_{1}-x_{0}) $$

results in

62 $$ U(x_{1},t_{1}|x_{0},t_{0}) = \Biggl(1+\sum_{n=1}^{\infty}\frac{1}{n!} \biggl(-\frac {\partial}{\partial x_{1}} \biggr)^{n} \bigl\langle \bigl(x(t_{1})-x_{0} \bigr)^{n}\bigr\rangle _{x(t_{0})=x_{0}} \Biggr)\delta(x_{1}-x_{0}). $$

The probability density function $p(x,t)$ obeys

63 $$ p(x,t+\Delta t) = \int U\bigl(x,t+\Delta t|x',t\bigr) p \bigl(x',t\bigr)\,dx'. $$

Inserting (62) gives

$$ p(x,t+\Delta t) = \Biggl(1+\sum_{n=1}^{\infty}\frac{1}{n!} \biggl(-\frac {\partial}{\partial x} \biggr)^{n} \bigl\langle \bigl(x(t+\Delta t)-x\bigr)^{n}\bigr\rangle _{x(t)=x} \Biggr) p(x,t). $$

Expanding $p(x,t+\Delta t)$ in a Taylor series in Δ*t* gives

$$ \frac{\partial p(x,t) }{\partial t} \Delta t= \sum_{n=1}^{\infty}\frac {1}{n!} \biggl(-\frac{\partial}{\partial x} \biggr)^{n} \bigl\langle \bigl(x(t+\Delta t)-x\bigr)^{n} \bigr\rangle _{x(t)=x} p(x,t) +O \bigl(\Delta t^{2}\bigr). $$

In the limit $\Delta t\rightarrow0$ we obtain the Kramers–Moyal expansion

$$ \frac{\partial p(x,t) }{\partial t} = \sum_{n=1}^{\infty}\frac{1}{n!} \biggl(-\frac{\partial}{\partial x} \biggr)^{n} D_{n}(x,t) p(x,t) +O\bigl(\Delta t^{2}\bigr), $$

where the *jump moments* are defined by

64 $$ D_{n}(x,t)=\lim_{\Delta t \rightarrow0} \frac{\langle ( x(t+\Delta t) - x )^{n}\rangle}{\Delta t} \bigg|_{x(t)=x}. $$

As long as these limits are convergent, then it is relatively easy to see that only connected Feynman graphs will contribute to the jump moments. In addition, we can define $z=x-y$, where *y* is the initial condition, $\tilde{z} = \tilde{x}$ and use the action $S[z(t)+y, \tilde {z}(t)]$. This shift in *x* removes the initial condition term. This means we can calculate the *n*th jump moment by using this shifted action to compute the sum of all graphs with no initial edges and *n* final edges (as in Fig. 1d for $n=2$).

As an example, consider the Ito SDE (3). From the discretization (6), where $h=\Delta t$, it is found that

65 $$ \lim_{\Delta t \rightarrow0} \frac{\langle ( x(t+\Delta t) - x )^{n}\rangle}{\Delta t} \bigg|_{x(t)=x} = \lim _{\Delta t \rightarrow0}\frac{ \langle ( f_{i}(x,t)\Delta t - g_{i}(x,t)w_{i}\sqrt{\Delta t} )^{n} \rangle}{\Delta t} , $$

which yields $D_{1}(x,t)= f(x,t)$, $D_{2}=g(x,t)^{2}$, and $D_{n}=0$ for $n>2$. Thus for the Ito SDE (3), the Kramers–Moyal expansion becomes the Fokker–Planck equation,

$$ \frac{\partial p(x,t) }{\partial t} = \biggl(-\frac{\partial}{\partial x} D_{1}(x,t) + \frac{1}{2}\frac{\partial^{2}}{\partial^{2} x} D_{2}(x,t) \biggr)p(x,t). $$

We have $D_{n}=0$ for $n>2$ even though there are nonzero contributions from connected graphs to these moments for $n>2$ in general. However, all of these moments require the repeated use of the vertex with two exiting edges; this will cause $D_{n} \propto\Delta t^{m}$ for some $m > 1$ and thus the jump moment will be zero in the limit.

We can envision actions for more general stochastic processes by considering vertices which have more than two exiting edges, i.e. we can add a term to the action of the form

$$ S_{V}[x,\tilde{x}] = \frac{1}{n!}\int dt \tilde{x}^{n} h(x) $$

for some *n* and function $h(x)$. This will produce a nonzero $D_{n}$. The PDF for this kind of process will not in general be describable by a Fokker–Planck equation, but we will need the full Kramers–Moyal expansion. If we wished to provide an initial distribution for $x(t_{0})$ instead of specifying a single point, we could likewise add similar terms to the action. In fact, the completely general initial condition term is given by

$$ S_{\mathrm{initial}}\bigl[\tilde{x}(t_{0})\bigr] = - \ln Z_{y}\bigl[\tilde{x}(t_{0})\bigr], $$

where $Z_{y}$ is the generating functional for the initial distribution. In other words, the initial state terms in the action are the cumulants of the initial distribution multiplied by the corresponding powers of $\tilde{x}(t_{0})$.

Returning to the Ito process (3), the solution to the Fokker–Planck equation can be obtained directly from the path integral formula for the transition probability (61). Let $\ln Z[J]$ be the cumulant generating function for the moments of $x(t)$ at time *t*. It can be expanded as

$$ Z_{\mathrm{ CM}}[J]=\exp \biggl[\sum_{n=1} \frac{1}{n!}J^{n}\bigl\langle x(t)^{n} \bigr\rangle _{C} \biggr], $$

yielding

$$ p(x,t) = \frac{1}{2\pi i}\int dJ e^{-J x} \exp \biggl[\sum _{n=1}\frac {1}{n!}J^{n}\bigl\langle x(t)^{n} \bigr\rangle _{C} \biggr]. $$

For the Ornstein–Uhlenbeck process the first two cumulants are given in (15) and (16), yielding (assuming initial condition $x(t_{0}) = y$)

66 $$ p(x,t)= \sqrt{\frac{a}{\pi D(1-e^{-2a(t-t_{0})})}}\exp \biggl(\frac {-a(x-ye^{-a(t-t_{0})})^{2}}{D(1-e^{-2a(t-t_{0})})} \biggr). $$

## Further Reading

The methods we introduced can be generalized to higher dimensional systems including networks of coupled oscillators or neurons [16, 17, 19, 21–24]. The reader interested in this approach is encouraged to explore the extensive literature on path integrals and field theory. Bressloff [13] covers the connection between the path integral approach and large deviation theory. The reader should be aware that most of the references listed will concentrate on applications and formulations appropriate for equilibrium statistical mechanics and particle physics, which means that they will not explicitly discuss the response function approach we have demonstrated here. For application driven examinations of path integration there is Kleinert [11], Schulman [34], Kardar [27] and Tauber [12]. More mathematically rigorous treatments can be found in Simon [35] and Glimm and Jaffe [36]. For the reader seeking more familiarity with concepts of stochastic calculus such as Ito or Stratonovich integration there are applied approaches [3] and rigorous treatments [37] as well. Zinn-Justin [26] covers a wide array of topics of interest in quantum field theory from statistical mechanics to particle physics. Despite the exceptionally terse and dense presentation, the elementary material in this volume is recommended to those new to the concept of path integrals. Note that Zinn-Justin covers SDEs in a somewhat different manner from that presented here (the Onsager–Machlup integral is derived; although see Chaps. 16 and 17), as does Kleinert. We should also point out the parallel between the form of the action for exponential decay (i.e. $D = 0$ in the OU process) and the holomorphic representation of the harmonic oscillator presented in [26]. The response function formalism was introduced by Martin et al. [29]. Closely related path integral formalisms have been introduced via the work of Doi [5, 6] and Peliti [7] which have been used in the analysis of reaction–diffusion system [8–10, 30]. Uses of path integrals in neuroscience have appeared in [14, 15, 17–21, 23, 25].

## Footnotes

1 This derivation is, strictly speaking, incorrect because the delta functional fixes the value of $\dot{x}(t)$, not $x(t)$. It works because the Jacobian under a change of variables from $\dot{x}(t)$ to $x(t)$ is 1.

2 This is also a manifestation of the normal-ordering convention chosen for the theory. Zinn-Justin [26] refers to this as the “$\epsilon(0)$ problem.”
